# An examination of motivation factors driving investor behaviours towards socially responsible community energy initiatives

**DOI:** 10.1016/j.heliyon.2024.e27490

**Published:** 2024-03-06

**Authors:** Helen Mullen, Charles Turkson, Adolf Acquaye

**Affiliations:** a Sustainability Monitor, Innovation Centre, University Road, Canterbury, UK; b School of Business, University of Dundee, Dundee, UK; c Department of Management Science and Engineering, Khalifa University, Abu Dhabi, United Arab Emirates

**Keywords:** Energy community, Socially responsible investments, Investor behaviour, UK

## Abstract

Community energy initiatives play a significant role at the grassroots level in the transition to Renewable Energy Communities and a low-carbon economy. However, these initiatives are hampered by multiple barriers at the market, institutional, organisational, and individual level. Funding cuts of state-supported feed-in tariff (FiT) policy in major markets such as Germany, Japan, China and the additional capping of the number of new installations that could be accredited under the FiT scheme in the UK. In light of these market changes and the need to accelerate the development and growth through the creation of new and/or complementary future community energy models consisting of private investors, a detailed understanding of the dynamics of community energy investor characteristics and socio-psychological motivations is increasingly important. First, a review is conducted including the theories that underpin and explain the factors that affect investor behaviour, after which a conceptual framework to examine investor behaviours towards socially responsible community energy initiatives is developed. The framework is used as the basis to construct and administer a survey involving sampling of 295 UK investors in community energy initiatives and the subsequent statistical analysis of the survey data and discussions of the findings. The results first capture the differences among investors with differing regional affect and investment behaviours. The study also provides the needed insight into better understanding the dynamics of investor characteristics and motivations of community energy initiatives. Results also indicate that investors are predominantly ethically-oriented, particularly toward environmental concerns. Additionally, community and social factors also appear to play significant roles in investor participation while financial orientation is least dominant.

## 1 Introduction

Community energy initiatives (CEIs) involving grassroots-level providers of renewable energy (RE) [1,2] forms part of the broader concept of Renewable Energy Community [3]. In addition to low-carbon energy provision, CEIs play a role in promoting environmentally-friendly consumption behaviours [4,5]. Seyfang et al. [6], define the concept, to denote “groups of individuals who voluntarily accept certain rules for the purposes of shared common objectives relating to energy; that is: (1) purchasing energy as collective groups (2) and/or managing energy demand and supply, (3) and/or generating energy.“.

CEIs share the same macro-level output, which is, renewable energy (RE) production. However, they vary considerably in purpose, structure, ownership and beneficiaries. This has led to the sector's pluralistic nature with multiple stakeholder types harbouring a range of motivations for their involvement. As a result, RE production is often a secondary outcome to other socio-economic motivations. Shareholders desire positive financial returns, while local residents may benefit from cheaper energy supplies. Regional development can also benefit the local labour market, economy and amenities, which in turn can generate fiscal revenues for local authorities [7]. Beyond financial and environmental gains, wider motivations include improved energy democracy^1^ [8], energy equity and other social benefits, energy security where communities are directly supplied [9] and aspirations towards degrowth^2^ [[10], [11], [12]].

Existing initiatives typically raised capital through a combination of loans, governmental and non-governmental grants, share- and bond-offers [13,14]. In Europe for instance, the ability to obtain cheap loans and attract share- and bond holders with competitive rates of return was largely underpinned by Feed-in-Tariffs (FiT) and tax-relief [15]. In fact, according to The International Renewable Energy Agency [16], FITs have been the dominant regulatory instrument in developed and developing countries alike, which has led to the growth of the renewable energy sector, including benefiting from the participation by community groups. In the UK, a shift in the economic landscape has, however, forcefully halted momentum in CEI growth. Government funding cuts to FiTs and the subsequent capping of the number of new installations that could be accredited under the FiT scheme each ‘tariff period’ preceded an 80% drop in new energy-related projects. Consequently, CEIs were most impacted as these changes rendered them financially unviable [17]. This is so despite CEIs being an important measure captured in wider policy initiatives such as the Green Deal [18,19]. Besides the UK, International Renewable Energy Agency [16] reports that there has been a fall in policy support in key markets such as China, Germany and Japan, which have all experienced cuts in government funding for FiTs from 2016. This notwithstanding, community energy organisations continue to adapt, show resilience and deliver value to local communities. Even during the COVID pandemic and lack of strategic, financial and political support, community energy continued to play a crucial role in contributing towards achieving net-zero in the UK through stakeholder engagement, increasing participation and embedding behavioural change [14]. While existing projects are protected, the shift in the economic landscape has compromised the financial structure for new projects, creating a need for alternative, innovative ways to raise capital [13,20].

What motivates share- and bond-holders to invest in CEIs can help explain their expectation of returns, especially where economic conditions can result in the cost of capital threatening the project viability. Academic literature indicates that investors of CEIs are motivated by a range of socio-psychological factors beyond financial gain. These factors include social norms, efficacy beliefs, social identity, and trust. The heterogeneous nature of those involved in ethical or collective endeavours contributes to these motivations [4,21,22]. Indeed, Naserisafavi et al. [23] note that understanding and reconciling diverse community perceptions can enhance sustainability efforts and support equitable governance. This is because values and expectations towards sustainability initiatives vary among people. In addition, a better understanding of what motivates investors to participate in green initiatives presents an opportunity to counter the negative impacts through effective and informed targeting and tailoring of incentives [24]. More targeted marketing could increase participation and engagement, depending on the characteristics of the investor base [25,26], whose characteristics and motivations can be better understood from studies such as this.

In light of these and the financial barriers facing community energy initiatives and the need to accelerate their development and growth through the creation of new and/or complementary future community energy models, this paper aims to examine investor behaviours as a source of funding for socially responsible community energy initiatives. Specifically, the objectives of the paper are two-fold.

1. To empirically assess the characteristics of existing investors in UK CEIs in order to understand the differences in the type, structure, stakeholders, beneficiaries and their localization to CEIs.
2. To provide fine-grained examination of the motivations and orientation driving investors of CEIs in order to better understand the dynamics of their value and materiality factors in order to potentially inform the creation of suitable incentives for future community energy models.

To address these issues, the rest of the paper is structured as follows: In the next section, the extant literature on CEIs on investor characteristics and motivations are reviewed including theories that underpin and explain the factors that affect investor behaviour. Following a review of the literature, a conceptual framework showing the inter-linkages between these motivations is developed and presented and a guide for subsequent assessment is presented in the methods section. This section also describes the data collection procedure and survey instruments. Thereafter, the results and analyses (Section 4) and discussions and implications of the study (Section 5) are outlined. Finally, the concluding remarks and directions for future research are noted in Section 6.

## 2 Literature review

### 2.1 What motivates community energy investment?

**Fig. 1:**
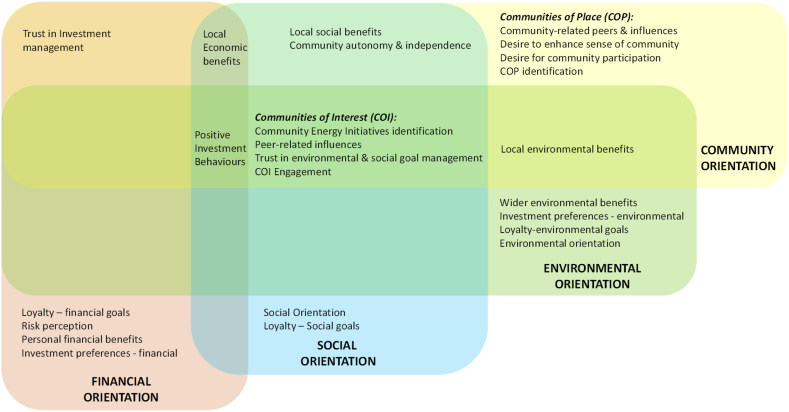
Conceptual framework developed for this study to examine of investor behaviours towards socially responsible community energy initiatives.

Literature shows a variety of factors that are believed to motivate investor interest in CEI investment [27]. These motivators could be potential financial rewards [28,29], whether the CEI has community-specific benefits [8,30], environmental benefits [22,[31], [32], [33]] and other social benefits transcending the investor's local community [26]. Indeed, CEI are aligned to the principles of environmental, social, and governance (ESG) model of developing and managing sustainability projects in society. In this section, literature on these motivators is discussed. More specifically, we show the inter-linkages between these motivators. These inter-linkages are also summarized in the conceptual framework in Fig. 1.

#### 2.1.1 Financial orientation

There is a general consensus in the literature on the role of risk, return on investment and other gain motives such as the price of energy supply in motivating CEI investments, though the consensus is absent on financial motivation as the dominating incentive. Bauwens [28] argues that CEI's institutional logic reflects the significance of the return motive, with Belgian market-oriented CEIs attracting greater numbers of financially-motivated investors, while community-gain motivations prevail in community-oriented organisations. This is supported by similar findings in Germany [32,34], Austria [35], and the Netherlands [25]. By contrast, some studies find financial motivations are present, but not dominant [29]. Holstenkamp and Kahla [32] go as far as to state personal financial gain may be the least important motivation.

Socially Responsible (SR) investments are largely considered to integrate non-financial drivers, such as ethical, social or environmental factors, into the investment process [36,37], thereby yielding both competitive portfolios and achieving better sustainable outcomes for society [38]. As CEI is a subset of SR investments, some similarities between them may be observed. Essentially, financial motives are present in SR investors but do not constitute the sole drivers [39]. For instance, Chung et al. [40] conclude that green fund investors “[seek] both superior financial performance and environmental consumption”. There seems to be a difference between the actual and perceived financial performance of SR investments. With regards to actual financial performance, Chung, Lee and Tsai [40] found that US-based green mutual funds tend to perform comparably to their conventional counterparts, and Chakrabarty et al. [41] suggest the same in exchange-traded funds across several national contexts. Amongst general investors in the UK, Barclays Bank [42] reported expectations of similar or greater returns from SR investments compared to general market rates, but those most interested held greater tolerance to risk. Conversely, Lewis [43] found that SR and non-SR investors similarly believed SR investments provide lower returns to market rates. A British industry report by Ethex [44] found that SR investors were more likely to accept lower returns in exchange for positive impact and to perceive SR investments as “too risky”, indicating that the difference lies in investors’ propensity to invest despite perceived lower returns and higher risk. Further, Lewis [43] observed expressions of guilt amongst SR investors who also held non-ethical (conventional) investments or savings.

The loyalty characteristics of SR investors appear equally mixed. By looking at green fund volatility, Chung, Lee and Tsai [40] provide evidence of greater loyalty afforded by SR investors, even during the economic crisis of 2008. Likewise, Chakrabarty, Lee, Singh, Alderson, Betker, Halford, Bharati, Doellman, Fu and Bouchet [41] support this finding in the US, though evidenced no greater loyalty in China, Canada or South Korea. Peifer [45] also confirms greater loyalty in SR investments, adding further that ‘dual investors’, holding both conventional and SR investments, display more loyalty toward the latter. A positive correlation emerged, however, between perceived poor performance and diminishing loyalty to SR investments. It was argued by Peifer [45] that greater financial orientation diminishes loyalty, while greater ethical orientation increases it. Such findings confirm the importance of financial incentives in SR, and the complications of investor characteristics and motivations. Indeed, even conventional investors are believed to make investment decisions beyond perceived financial risk and return alone. In the realms of behavioural finance, observations suggest investor motivations may pertain to various personal tastes and goals [46], positive affect towards a company, relating to the extent it aligns with investor identity and self-concept [47], while Statman et al. [48] note the positive influence of company-effect on investor perceptions of risk and reward. Such complications in investor motivations are reflected across the literature regarding CEI investors, covering a wide array of influential factors beyond financial incentives.

In relation to the risk of returns, traditional finance theory posits that sustainable investments, such as in CEI, would only be considered if they are equally attractive as other investments in terms of risk and returns [49,50]. However, from a sustainable investment point of view [51], motivations may be driven by factors beyond the risk of financial return since some studies; see for instance Belghitar et al. [52] and have provided evidence of higher risk of financial cost of sustainable investments, whiles others such as Humphrey et al. [53] and Sharma et al. [54] have reported that there is no compromise on return or risk in sustainable investments. In examining risk-return preferences for projects such as CEI, Curtin et al. [55] noted that although majority of citizens in the Irish market are motivated by financial attributes such as return, they are highly risk-averse. In Germany, Bauer and Menrad [56] highlighted the importance of annual risk-return for individual investors and Salm, Hille and Wüstenhagen [34] distinguished between “local patriots” and “yield investors”, whiles noting their different risk-return expectations.

#### 2.1.2 Community orientation

The literature casts a wide net over potential motivational influencers, and in varying combinations. Motivations to invest may be derived from the perception of local benefits a CEI may bring. Regional Added Value – the localization of economic benefits, is a recurrent theme which may be expressed through local employment [8,30], regeneration and socio-economic development [57], or energy security and independence from energy companies and rising tariffs [58]. Other community benefits could include community autonomy from external bodies (including government) and democratic control over local issues [59], and the desire to participate in or develop the community [8,31].

While Fleiβ, Hatzl, Seebauer and Posch [35] state that despite finding financial incentives the dominant motivator, cohesive communities are likely to afford less importance to these, the latter notion is also supported by Salm, Hille and Wüstenhagen [34]. Moreover, Walker et al. [60] found that greater cohesiveness facilitated acceptance and mobilisation of community energy projects. The extent to which a CEI is embedded in its community is, therefore, a factor of interest. A cohesive community underpinned by a social network is correlated with the level of shared norms, values and trust and engage in purposeful actions [61]. Bauwens [28] shows how the extent to which a CEI is embedded in the community is reflected in its institutional nature, as its structure and organisation is shaped by social norms, trust and social identification of community actors. Where institutional logic is community-oriented, community investors are more likely to be attracted, while market logic will attract financially-oriented investors.

The extent to which each community factor motivates an investor has been linked to individual, community, regional and institutional factors. Literature covering the socio-psychological perspective proffers the degree to which investors identify with the cause as a significant factor. Bamberg, Rees and Seebauer [26] for instance, posit that low-identifiers tend towards cost-benefit analysis when deciding whether to participate, with costs including time, effort and money. Conversely, high-identifiers are more likely to share interests, norms and goals with their community project, rendering participation an “inner obligation”. This is congruent to Bauwens’ [28] finding that investors with greater social identification were inclined to participate in absence of material benefits. Thus, motivation may be due to closer alignment between individual and collective values and goals, creating a “parallel rationality” to participate [62].

Social norms and trust are offered as mechanisms to explain social identification and propensity toward community energy participation. Social norms are “expected forms of social behaviour, based on (…) implicit social rules [which] exercise a coercive influence” [63]. In this case, these social expectations pertain to environmental concerns and behaviours [26,64,65], or community-related concerns [25,59]. Peers, such as community actors, shape norms and expected behaviours, which may encourage greater participation, either through a desire for external validation from an individual's network, or avoidance of socially-imposed sanctions [62]. In addition, shared orientations may foster greater trust; a prerequisite for investment and collective action [60,66]. Kalkbrenner and Roosen [64] posit trust and social norms as the strongest associations with a willingness to participate, while Bauwens [28] notes the significant relevance of both.

Other distinctions in the literature regarding community actors pertain to Communities of Interest (COIs) and Communities of Place (COPs). The former encapsulates more geographically-disparate parties with shared interests, i.e. communities united by social movements, such as pro-environmentalism, while the latter captures bonds between parties because of where they reside or spend a lot of time. The strength of social networks in effecting CEI investments in either community type may be linked to word-of-mouth effects [29,58]. Becker et al. [67] state dominance in communities of place in the community energy literature. Similarly, Bauwens [28] found that social norms were more prevalent in communities of place than in communities of interest, stimulating greater investment rates compared with non-local projects.

#### 2.1.3 Environmental orientation

Bauwens [28] reports that organisations with greater environmental purpose attracted investors with correlating pro-environmental orientations as defined by behaviours and self-identification, and greater social identification with the organisation. Such investors placed greater value on renewable energy production than material incentives compared to more market-oriented counterparts. Radtke [31] and Holstenkamp and Kahla [32] cite environmental incentives as a dominant motivator. Other research identifies environmental concerns as a significant but not dominant factor, coming behind social-norms and trust [64], amongst other normative goalframes such as community- or social benefits but above personal gain incentives [68], or closely behind gain-goal frames [25,58]. Similarly, in socially responsible investors, amongst a range of social and environmental issues, environmental incentives were significant [43,69] or dominant [44]. Notably, however, a distinction must be made between orientations, investment intentions and behaviour. In the case of renewable microgeneration, Balcombe et al. [70] observed that environmental motivation was significantly displaced by financial concerns once costs were revealed.

#### 2.1.4 Social orientation

A person's dospisition has a role in setting the intention to engage in a behaviour: Environmental concern partly elicits pro-environmental behaviours [71], or intention to invest [58,64,72], which may also result from a positive behaviour towards renewable and community energy [58,64,72], or investing in innovative institutions (for instance, less developed CE models) [28].

A number of theories provides a theoretical base to explain factors that affect investor behaviour. Lindenberg and Steg [71] for instace refer to Goal-Frame Theory to explain the role of pro-environmental behaviours. Such behaviours are linked to a dominant normative goalframe, which compels an individual to “act appropriately”, while gain and hedonic goalframes orient one to “guard and improve (…) resources”, or to immediately “feel better” respectively. Where gain goalframes dominate, such as in financially-oriented investors, the authors use the Theory of Planned Behaviour to explain gain-related motivation in engaging in pro-environmental behaviours: behavioural intention results from a cost-benefit analysis regulated by intentions towards the behaviour, social pressure to perform it (social-norms) and perceived ability to perform it. However, pro-environmental behaviours are typically linked to a normative goalframe; here the authors posit Value-Belief-Norm theory, where greater awareness of the personal impact on environmental issues and exposure to the environmental social-norms of others in society, and a belief their behaviour will effect change, diminishing the influence of gain and hedonic goalframes. Thus, the motivation to avoid the costs of pro-environmental behaviour is weakened [71]. This may reflect in Barclays Bank [42] SR investment strategy, which accounts for greater social orientation in potential SR investors in overcoming the discomfort of investing. Additionally, Bamberg, Rees and Seebauer [26] highlight the potential temporal-causal effect of CEI participation, where exposure to CE actors causes the internalisation of group norms and efficacy beliefs to produce greater social identification with environmental causes, thus shifting initial gain-orientations towards normative orientations.

Fleiβ, Hatzl, Seebauer and Posch [35] emphasise the distinction between desire and belief in determining motivations, asserting that both are necessary to move beyond intent. However, despite finding the presence of belief in CEIs to achieve one's pro-environmental goals, belief in achieving personal financial goals ultimately motivated investors.

### 2.2 Contextual factors

Investor motivations for CEIs may be influenced by contextual factors. These context-related factors may result in differences in how these investor orientations affect the investor motives towards CEI investment. These contextual factors are reviewed in this section.

#### 2.2.1 Institutional characteristics

Studies find a link between investor orientations and institutional characteristics such as RE technology and institutional innovation. Bauwens [28] finds innovators and early adopters of institutional innovations (such as CEIs) are more idealistic and norm-driven, while more gain-oriented early-majority investors wait for reassurance through proven technologies and greater mainstreaming of community energy models. Similarly, Holstenkamp and Kahla [32] document more established CEIs with historically good financial returns in Northern Germany, along with greater return motives in projects in that region.

#### 2.2.2 Regional characteristics

Few studies examine regional differences in investor types, though regionality appears linked to community energy presence and form. In 2013, the UK's CEIs were disproportionately located in Scotland, South-East and South-West England, with indications of being predominantly rural [6,73]. Holstenkamp and Kahla [32] note differences in institutional logic as a result of socio-economic variables in different regions of Germany, with less affluent Eastern regions opting for models with greater regional-added value over personal-gain incentives. Further, Moss et al. [74] posit regional differences across Germany affect the uptake of community energy; a consequence of local history and the interaction of socio-economic, structural and socio-political factors across formal^3^ and informal^4^ institutions. Rather than proactively pursuing RE – a stance fostered by a greater social movement presence in Berlin, poorer areas may reactively mobilise to defend against external investors profiting from their community resources, seeking community energy models to provide regional-added value [74]. Similarly, in Scotland, Bomberg and McEwen [59] discovered CEIs in deprived communities could facilitate enough support where shared goals were aligned, for instance, with the purpose of regeneration and alleviating fuel poverty. It appears socio-economic status may not dictate the presence of CEIs but rather how they are organised and which goals they pursue.

### 2.3 Conceptual framework

The conceptual framework as presented in Fig. 1 consists of four overlapping motivators, derived from emergent themes in the literature on the nature of CEIs, community energy and socially responsible investments and those engaging with these alternative investments. The framework represents a collection of factors to explain why an individual is motivated to make a community energy investment. These factors are organised into categories according to overarching attributes: financial, community, social and environmental orientations. Investors were assessed against each relevant factor to determine the extent of orientations within each category. Consequently, this framework should identify any differences between investor types and provide the theoretical underpinning to help explain them. Additionally, differences along the contextual dimensions are also explored in order to understand whether institutional and regional factors cause a difference in investor motivations.

## 3 Methods and measures

### 3.1 Scope of the study

The CE landscape is made up of CEIs affiliated with umbrella organisations, in addition to independent initiatives. In the UK, Mongoose Energy, which was taken over by Bright Renewables in 2019, represented such an umbrella organisation, with ten associated Community Benefit Societies (CBS), with each overseeing between one to five CEIs. Of the ten CBSs, five agreed to participate in this study. The company engages in identifying, developing, financing, building, and managing community-owned, clean energy projects including wind and solar projects. The research subjects are CE investors from each participating CBS. While not all CBSs are involved in the study, there was considerable cross-over with other Mongoose Energy CBSs and beyond, as many investors held more than one investment.

### 3.2 Data collection

Drawing on the themes in the literature and findings from sector-specific community energy and socially responsible research, a theoretical framework was formulated for the study. This then formed the survey structure, focusing on the data required to assemble investors’ motivational profiles and test against the underlying theoretical constructs. Consequently, an online survey was used in collecting the necessary responses for the assessment.

A number of umbrella organisations makes up the CEIs in the UK. Given that the sample for the study consisted of investors belonging to only Bright Renewables, the study therefore assumes a multistage sampling method consisting of first, cluster sampling and secondly, voluntary sampling. The study first assumes that, the whole population of CEIs in the UK is divided into subgroups or clusters, one of which is Bright Renewables. From this subgroup or cluster, which is assumed to be characteristic of all CEIs in the UK, a voluntary response non-probability sampling of individual investors belonging to the population of Bright Renewables investors are sampled for the study.

Overall, 446 respondents clicked through to the survey from a total population of 1634. This sample size is an appropriate sample size for the study, given the total population per Yamane formula [75] as confirmed below.

The Yamane formula is given by Yamane [76]:

n=N1+N(e)2

Where:

n = The sample size of the study to be determined

N = Population of study (the number of investors belonging to the five Community Benefit Society of Bright Renewables at the time of the study)

e = Level of significance, which is assumed to be 5% (0.05) in this study.

Given that N=1634 investors, and e=0.05, solving for n (appropriate sample size) gives:

n=N1+N(e)2=16341+1634(0.05)2

Hence.

n=321.34≈322 investors.

Given that 446 respondents in the survey is greater than 322, the statistically significant inference can be drawn from the sample study.

In cleaning up the survey data, non-consenters, invalidated responses through a technical glitch, and early abandoners were removed, leaving a sample of 295 respondents. This represents a sample of about 18% of the population or a calculated level of significance of 5.27% (or 0.0527) or 94.73% Confidence Interval for the given population and per the Yamane formula. Early abandoners typically abandoned, most frequently at the question regarding investment-type (bonds and/or shares) and achieved only, on average, 22% progress in answering the survey questions. The data gathered is used to make inferences on the population of Mongoose Energy investors, regarding investors’ characteristics, orientations and incentives. Before collecting data from the survey participant, ethical approval was granted by the Kent Business School, University of Kent Research Ethics Committee. In addition, informed consent was obtained from all respondents prior to their participation in completing the questionnaires.

### 3.3 Survey Design and measurement

The survey consisted of four main sections categorised as community energy investment section, financial section, demographic section and ethical section. A summary of the survey structure is provided in the Appendix. The summary provides an overview of all the variables gathered under each section and the levels and units of measurement.

The community energy investment section is mainly related to the investment profile of respondents and the factors influencing these investment decisions. Exploring regional-affect relied on the respondent's view of CEI-locality in relation to them, rather than geographical identification. Respondents were asked if they considered their first CEI as very local, close enough to be deemed local, or in a place of attachment (a community they identify with but do not inhabit, such as a childhood community). These ‘local investors’ (LIs) were identified as subject to regional-affect, whereas non-local investors (NIs) were identified as having no attachment or regional connections.

Also, influences relating to beneficial outcomes were drawn from themes in the literature specific to CEIs and CEI investors. Recommendation influences were informed by literature concerning socio-psychological effects on investment behaviours, COP and COI influences, such as word-of-mouth and social norms. Offering a range of influences across each framework orientation allowed investors to rate influential levels relative to each other. Multi-investors (MIs) were asked to focus on first investments, then repeated these questions for subsequent investments. As CEIs are a form of ethical COI, this was to ascertain the socio-psychological effects of exposure to CEI group norms over time [26].

The financial section sought information about investors’ financial influences, risk perception and investment behaviour. The sensitive nature of personal wealth necessitated banded values regarding net income, community energy, socially responsible and conventional investments and savings (IS), and opt-outs were also provided. While these measures reduced data granularity, they minimised the risk of abandonment. Fully assessing risk appetite requires numerous questions and would not ascertain how investors perceive community energy compared to alternative investments. However, in this study, we compare risk perception on community energy and socially responsible investment compared to conventional investment. This comparison is key to exploring why an investor might invest, and whether investors participate to benefit *from* comparative risk perceptions, or *in spite of* them. This utilised a 6-point rating scale, forcing distinctions between high or low risk.

The ethical section was much more broad measuring investor loyalty to investment, social, environmental and community identification as well as their intentions on future investment. Community energy models issue withdrawable shares, so share exchange facilities do not apply [77]. Investors are typically long-term and anticipated to remain invested over the project lifetime. To assess loyalty accordingly, investors were asked about discouragement from future community energy investments rather than addressing loyalty to existing community energy investments. Though this presents less favourable measures in the form of intent rather than behaviour, as per the literature [45], it is an appropriate equivalent in the community energy context.

Social and environmental orientations were measured using the Ethically Minded Consumer Behaviour scale (EMCB) developed by Sudbury-Riley and Kohlbacher [78]. The EMCB holds several advantages, including validity and reliability. Moreover, it focuses on the UK population (the context of this study) and it has strong predictive quality in identifying respondents who engage in social or environmental causes and is robust to social desirability bias [78]. This latter point is pertinent given these orientation variables are likely the most susceptible to social desirability bias. Furthermore, it produces reliable results with only 10 questions, minimising survey fatigue. Besides that, two recycling-based questions were amalgamated into one, and another was added to assess if respondents engaged in less mainstream environmental behaviours.

Community identification scales were adopted from peer-reviewed studies. CEI identification was measured using three questions from Bauwens' [28] ‘social identification’ scale except for two questions which were removed based on similarity and concerns regarding survey fatigue. To measure COP identification, van Vugt's [79] measures were used. Testing for Cronbach's Alpha, both scales meet reliability requirements. As for community engagement, it was measured in several ways, including peer recommendations and peers interested in participating and ethical COI engagement.

Finally, the demographics section sort to gather information about the individuals participating in the survey. The questions in this section bothered on qualification levels [80], occupational status [81], preferred newspapers [82], among others. Employment status was modelled from ONS [83], but simplified following feedback.

### 3.4 Data analysis

The survey instruments gathered various data types. Likert-scale data were assessed using medians for single items, and means when aggregated onto scales. Reliable and valid scales were adopted where available. However, the financial influences scale was purposed for this research. Reliability testing returned a Cronbach's alpha of 0.673. In this study, a range of non-parametric (Mann-Whitney U and Wilcoxon matched-pair signed-rank for independent and related samples respectively), and parametric (independent samples *t*-test) tests were used where appropriate to draw inference about the statistical difference. Similarly, correlation analyses were accordingly based on Pearson's Coefficient for scale data and Spearman's Rank Order for item data.

Data corresponding to framework orientations were statistically tested against investor groups: local and non-local, single and multi-investor. Where statistical differences were presented, respective group values were reported (means, medians or proportions). Otherwise, groups were equally awarded total population values. This identified where investor groups differ in orientation and to what degree. Variable values were awarded to all relevant categories to accommodate overlapping structures. Values differed in measurement scales, so these were linearly standardised to ensure proportional values were attributed to each orientation.

## 4 Findings and results

In this section, a description of the respondents, in terms of their region characteristics and portfolio type, is first provided. This is followed by an analysis of their demographic, financial and ethical profiles. This provides the basis to examine the influences on CEI in subsequent parts of the section. Throughout, an examination is made based on the investor groups: single investors (SI), multi-investors (MI), local investors (LI), and non-local investors (NI). The investor types were categorised based on their investment behaviours (singular vs. multiple investments) and the CEI location (local vs. non-local investors).

### 4.1 Profile of respondents

#### 4.1.1 Region and Investment portfolio

Table 1 presents the distribution of respondents across the various groupings. When aggregated, MI and LI make up the dominant behaviour and regional characteristics at 59% and 55% respectively. Multi Non-Local (MNI) is the dominant investor by behaviour and region at 32%, with SNIs producing the smallest subset at 13%, as shown in the table below.

**Table 1:** Count and sample proportions of investors by locality and number of investments.

Investor Type	Count	Percentage
**Single Local**	82	27.80%
**Single Non-Local**	38	12.88%
**Multi Local**	81	27.46%
**Multi Non-Local**	94	31.86%

#### 4.1.2 Demographic distribution

Most respondents were male (70%) and aged 51–80. Moreover, newspaper readership was primarily left-leaning at 65%, with right-wing newspapers contributing 22%. The prevailing employment status is retired at 57.7%, followed by full-time employed (16.4%) and self-employed (14.4%). As for occupational level, top-level managerial, professional and administrative occupations are overwhelmingly the dominant categories (81.1%), with ‘intermediate’ as the modal equivalent at 43%. This distribution holds across all groupings. In addition, 89% of respondents reported qualifications to a minimum of degree or equivalent professional-level. As for net income, this sample indicates a median net-income of £30,000-£39,999, with a negative skew indicating income is predominantly less than £40,000.

Statistically significant demographic differences between the subgroups pertain to age, news-readership and qualification levels, with SIs showing a slightly more pronounced skew towards older age compared to MIs. While MIs vary through higher readership of centrist newspapers, LIs being twice as likely to read right-wing newspapers than NIs. Finally, SIs make up a smaller proportion of those with a minimum of degree or professional-level qualifications, compared to MIs.

#### 4.1.3 Financial orientation

Respondents reported on two different classes of investments they partake in; firstly, their total Investments and Savings (IS) and secondly, their investments in Socially Responsible (SR) CEIs investment. 60% of IS investors reported that their investment totalled over £100,000, whiles a median of the sample reported a total IS investments of £50,000-£100,000. These excludes investments in CEIs. On the other hand, these same investors reported that their modal range of investments specifically for socially responsible CEIs was between £1000-£5000. Respondents indicated they would likely invest in CE again. This inclination appeared stronger for CEs holding regional affect (Medians: local - 3.29 and 2.96, non-local - 2.61). Moreover, median values showed respondents were ‘slightly likely’ to avoid further investments if existing CE investments underperformed against financial expectations.

CE (and SR) investments were typically perceived as less risky than conventional counterparts. CE investments are seen on average as 16% and 3% less risky for shares and bonds respectively. With very low skewness, distributions were deemed ‘fairly symmetrical’ (Bulmer 1979, 63) so mean values were used to represent these data for a more detailed picture.

Of personal financial factors concerning the decision to invest, respondents were typically more influenced by return of investment (ROI) and tax relief.

Statistical analyses indicate SIs and MIs differ by likelihood for further CE investment, several financial influences and comparative risk perception regarding shares.

With regards to the likelihood of future investment, Medians for both SIs and MIs indicated they were ‘somewhat likely’ to make another CE investment regardless of location, though on average, MIs were more likely to indicate a preference for local CEIs. On the other hand, future CE investments were overall more likely for NIs. Medians returned ‘somewhat likely’ for both regarding CEIs with regional-affect, though LIs indicate lower inclinations toward non-local CEIs.

MIs valued financial influences more, with median of 1.5 compared to 1.35 for SIs. When measured against other financial factors, SIs place more emphasis on confidence in CE investment management and local economic benefit*s.* A statistical difference was found for the comparative risk of shares only: SIs perceive CE investments as lower risk than similar conventional alternatives.

#### 4.1.4 Ethical orientation

Average scores for social and ethical orientations were similar across all investor types and regions, with the most typical response of ‘mostly true’ reported for ethical behaviours. As such, orientations yielded no significant differences between groupings. Statistical differences were produced for SR investment behaviours and some local benefit influences only, otherwise, ethical profiles appear reasonably similar.

The mean response for social orientation presented at 3.87 compared to 3.66 for environmental orientation. Referring to peers with similar ethical concerns, this sample tends towards more frequent engagement in ethical COIs. Again, responses were very similar across all groupings with no statistical differences. Of all investors, 70% hold positive savings accounts and/or investment and of these, 65% became SR investors before investing in CE.

Another indication of environmental orientation involves preferences expressed for opportunities to invest in riskier CEIs with unproven, immature low-carbon technology. Compared against investors expressing predominantly financial preferences, 45.7% requested this opportunity. COP identification could only be applied to those categorised as local. Of these, a mean of 0.44 on the COP identification scale puts identification closer to neutral, with a slight tendency towards positive identification.

All respondents were asked to rate their identification with their respective CEI(s), which is a form of community of interest each has in common. In contrast to COP identification, a stronger relationship is evidenced with CEIs, with the mean presenting at 0.72, placing the average response closer to ‘somewhat agree’. Knowledge of other interested investors relates to those known within COP or COI for LI and NIs respectively. 44% of investors knew at least one other person interested in participating in their CEI.

**Fig. 2:**
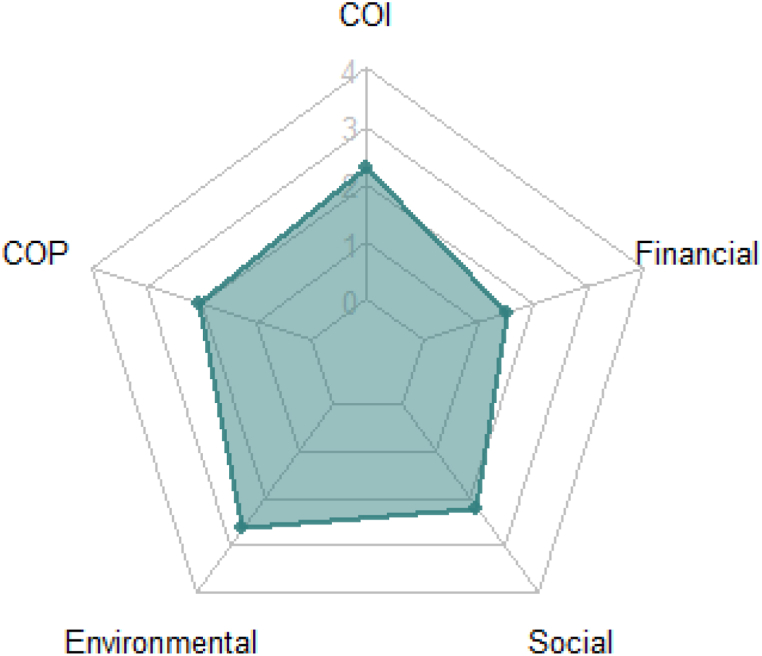
Affinity of All Investors to each Framework Orientation Category.

Each finding discussed was organised into the framework to assess the extent investors were drawn toward each orientation category. Respondents indicated the most pronounced orientation underpinning their CE is environmental. Social is the next most significant orientation, though this is very closely followed by COI. Financial orientation presents at fourth, while COP is the least strong. If COI and COP factors are aggregated into an overarching ‘community’ orientation, financial is relegated to the lowest position (see Fig. 2).

**Fig. 3:**
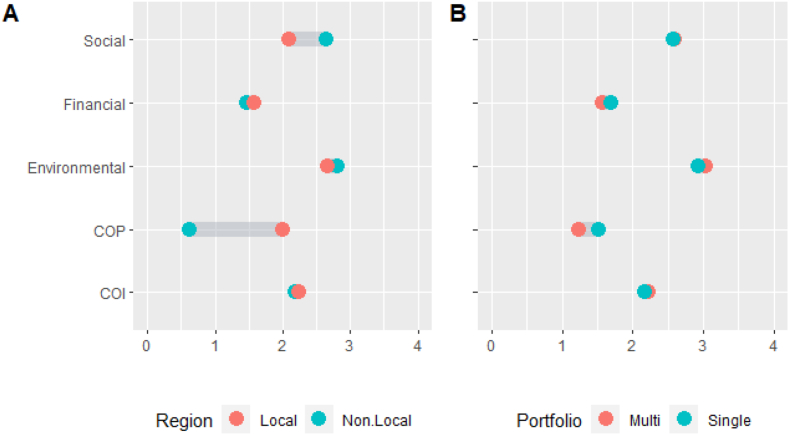
An overview of the affinity of investors by Region (3A) and Portfolio (3B) type to each framework orientation category. Fig. 3A highlights the differences between Local Investors (LI) and Non-Local Investors (NI) with respect to their motivations driven by the following orientations: Community (COP and COI), Financial, Social, Environmental. Fig. 3B highlights the differences between Multi Investors (MI) and Single Investors (SI) with respect to their motivations driven by the following orientations: Community (COP and COI), Financial, Social, Environmental.

In terms of sub-differences, as shown in Fig. 3, SIs and MIs expressed statistically significant differences regarding SR investment behaviour and in preferences for unproven RE in future CEIs. At 80%, NIs are more likely to hold more SR savings and/or investments than LIs (63%). Neither proportions of investors holding these prior to their CE investment, nor those expressing preferences for unproven RE technology CE investments were statistically significant.

Though equally likely to hold positive savings and/or investments as per total sample proportions, at 75%, MIs make a much higher proportion of those who held these prior to their CE investments (41%), compared to SIs (25%). Also, MIs were more likely to express a CE preference for unproven RE technologies in future, while appearing to identify much less towards their COPs, holding a mean of 0.17 compared to SIs’ 0.72.

LIs present a higher mean at 0.826 compared to NIs' 0.60, indicating LIs hold a stronger association with their CEIs. NIs were not asked about COP so a comparison cannot be made, but for reference, LIs’ means were 0.444. Moreover, LIs were more likely to know others also interested in participating in their first CEIs.

In order, NIs present the most pronounced environmental and social orientations as highlighted in Fig. 3A. In addition, environmental is the strongest orientation for locals, followed by COI, social, COP and financial. NIs hold a lesser financial-orientation, though it ranks low for both groups (Fig. 3A).

As shown in Fig. 3B, overall, both investor types display similarities with the biggest differences relating to COP, financial and environmental orientations. Both have prevailing environmental orientations. Social orientation is second for SIs, closely followed by COI, whereas these orientations present joint-second for MIs. As highlighted again in Fig. 3B, COI is more prevalent in MIs, though it is almost as strong for SIs, whereas COP is notably stronger for SIs. Financial orientation is greater amongst SIs but lowest ranked overall, whereas COP is the lowest ranked for MIs.

These discrepancies between the subgroups are summarized in the following figures.

### 4.2 CEI investment influences

When compared to other influences on CE investment, personal and broader financial factors appear less influential than environmental and trust-oriented factors. The most significant social influence overall is trust to manage ethical goals, though notably, wider environmental benefits are most influential over all categories. This was the only factor deemed a ‘significant influence’. Overall, more emphasis was placed on environmental factors than all other categories.

**Fig. 4:**
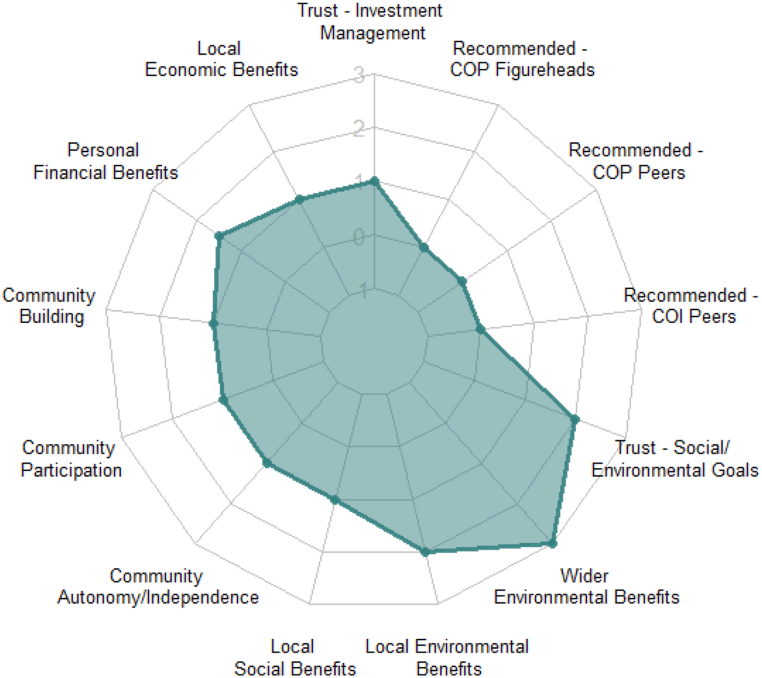
Influential Factors for CEI Investments (All Investors). NOTE: 1 Neg. influence, 0 No influence, 1 Slight Influence, 2 Moderate influence, 3. Significant influence.

Community influences in the form of recommendations are typically less valued than those within the other framework categories; COP and COI sources each have medians returning ‘no influence’. Community participation and building shared a median producing ‘slight influence’. Community participation excepting, community factors were not statistically significantly different between SIs and MIs. These tendencies have been summarized in Fig. 4.

In terms of sub-differences, testing for statistically significant differences, LIs and NIs differed only on future CE investments and local economic influences. Otherwise, both types follow the typical investor financial profile. Local economic benefit was the only statistically different financial influence but medians were the same.

**Fig. 5:**
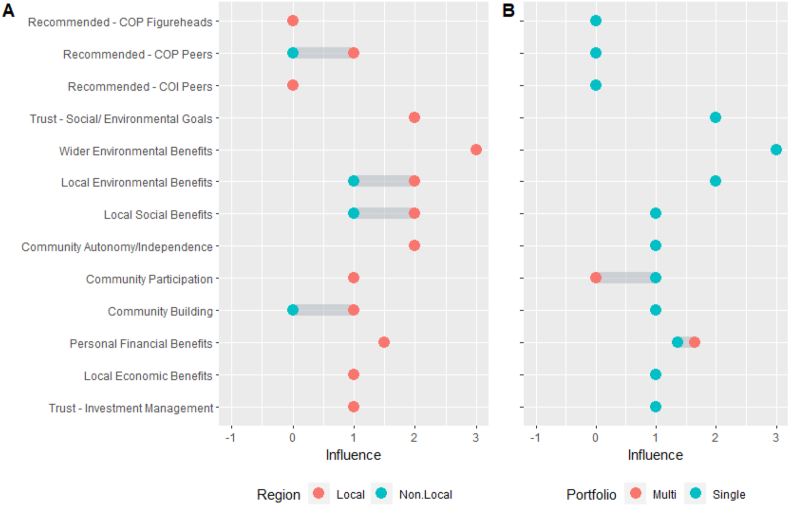
Influential Factors on CEI Investments for Investors by Region (5A) and Portfolio (5B) type. Fig. 5A highlights the differences between Local Investors (LI) and Non-Local Investors (NI) with respect to main levels of Investment influences for the category: Community, social and environmental influences. Fig. 5B highlights the differences between Multi Investors (MI) and Single Investors (SI) with respect to main levels of Investment influences for the category: Community, social and environmental influences.

Referring to Fig. 5A, LIs deem local social and local environmental benefits as more influential than NIs, while ratings for remaining social and environmental benefits did not reach statistical significance. Furthermore, LIs place more emphasis on recommendations from COP-peers than NIs, though the median for COP figurehead recommendation returns ‘no influence’ (Fig. 5A). Interestingly, medians for both LIs and NIs on community participation reflect a ‘slight influence’. However, LIs value community building more at ‘slight influence’. Referring further to Fig. 5A, NIs also place some value on local economic, environmental and social benefits, though not to the same degree as LIs. Community participation was valued more by SIs with a median indicating ‘slight influence’ compared with a median of ‘no influence’ by MI (Fig. 5B). Fig. 5B also highlights the fact that, MI placed more value of personal financial benefits as compared to SI.

**Fig. 6:**
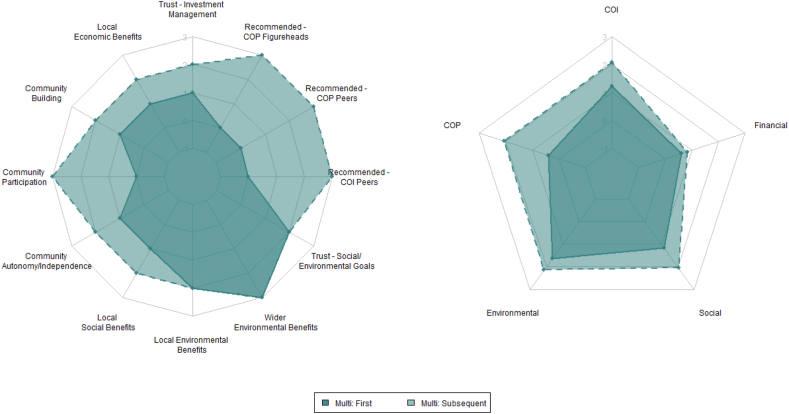
Factors influencing Multiple CE investment (first and subsequent investment). NOTE: 1 Neg. influence, 0 No influence, 1 Slight Influence, 2 Moderate influence, 3. Significant influence.

There was no statistical difference returned on recommendations from COI peers. The subgroup differences with respect to investor region and investor type are summarized in the spider charts in Fig. 6.

## 5 Discussion and implications of study

### 5.1 Demographic profile

Medians for all respondents indicate a higher-than-average net income for UK residents (£25,378 in 2017) [84], while total savings exceed third-quartile rankings, set at £46,000 in 2016 [85]. This is likely reflecting investment criteria, which avails CE investments to sophisticated investors and high-net-worth individuals only [86]. Pursuant to the sample's age demographic, older investors are more likely to have accumulated the wealth necessary to participate.

In terms of gender, wealth and qualifications, this demographic profile mirrors those of German counterparts [25,31,64,87], and almost the reverse of profiles in Norwich Transition Town [88]. Conversely, Ethex [44] represents another British context, with similar findings regarding gender and wealth. As women equally held other SR investments, this may indicate something unique to CE which commands significantly greater male participation.

### 5.2 Financial profile

#### 5.2.1 Investment distributions

Differences in distributions of CE, and other SR and conventional IS amounts run counter to comparative risk perceptions. Despite being perceived as lower risk, CE investments heavily tend towards lower amounts, whereas SR is normally distributed, and total IS skewed towards higher amounts. Risk perception measured investments, however, and does not refer to lower-risk savings vehicles likely included in total IS. Further, the Financial Conduct Authority (FCA) restricts CE investments to 10% of net assets for most investors, likely accounting for lower distributions and apparent discrepancies with risk perceptions.

#### 5.2.2 Investment loyalty

Overall, respondents state a slight inclination to abandon CE following financial underperformance. Interestingly, results indicate a homogenous response as no significant differences presented across groupings. Accordant with Peifer's [45] assertion regarding the diminishing effect of financial orientation on investment loyalty, this sample indicated similarities when correlated against financial influences. However, negative effects of ethical orientation on financially dependent loyalty were uncorroborated as no correlation was found. Though interesting indicators, these should be interpreted with caution, particularly as correlations were small and different measures of financial and ethical orientation were used across studies. In this instance, loyalty could not test behaviours in absence of a CE share-exchange and therefore facilitated a measure of intent instead.

#### 5.2.3 Risk perception

Interestingly, though several British studies indicate a relationship between higher risk tolerance and SR investors, this study finds SR investments are perceived as less risky than conventional investments. Further, respondents typically perceive CE investments as the least risky vehicle, despite heightened risks associated with CEIs noted in the literature [40,57,89,90]. These unexpected findings eliminated opportunity to test propensity to make CE and SR investments, in relation to perceived greater financial risks. These rankings were unanimous across groupings and when further disaggregated into behaviour-by-regional groupings.

#### 5.2.4 State-supported financial incentives

The cuts on state-supported financial mechanisms of FIT were rated ‘moderate’ and ‘slight’ influences respectively and were valued more by MIs than SIs. This suggests absence of these incentives may have implications for repeat investment behaviour, and to a lesser extent, investor recruitment. Additionally, as FIT broadly supported ROI as risk-reduction and return-enhancing mechanisms respectively, it should also be accounted for, along with its unanimous ‘somewhat influential’ ranking.

Correlations measured between propensity to abandon CE through financial underperformance and ROI and FIT were found present for SIs but absent for MIs. Disaggregating behaviours further by regional-affect, this absence remains for non-local multi-investors (MNIs), though their local counterparts indicate ROI is a factor (medium correlation). A near-identical ROI correlation is reflected with single local investors (SLIs). Single non-local investors (SNIs) correlate with FIT only (medium correlation)**.**

Abnormally high CEI numbers were rushed through to meet FIT cuts and capping deadlines in 2015, bringing attention to CE and these incentives. Between 1985 and 2016, over a quarter joined in 2015, and of MIs, regional numbers were comparable (circa. 20.6%), while SIs constituted 37.2% LI and 32.3% NI. This may partly explain SNIs’ correlation with FIT – potentially unlikely investors motivated by hype concerning the loss of incentives felt amongst COI actors. Similarly, heightened efforts to spread awareness of FIT within COPs may have persuaded otherwise indifferent individuals to seize the opportunity.

If we accept the underperformance variable as an indicator of financially-dependent loyalty to CE, the loss of state-supported incentives may explain both the recruitment and subsequent determent of many SIs. Reductions in ROI may also bear some adverse impact on numbers of locality-sourced investors. Overall, SI and/or LI loyalty seems somewhat sensitive to changes in the financial incentives structure, while MNIs appear insensitive.

Attempts to clarify the incentive characteristics responsible for eliciting differences in loyalty-responses proved elusive. No correlations were found between comparative risk perception and FIT, while controlling for net-income only slightly reduced the correlation between tax and financially-dependent loyalty. Though tax and net-income bear strong direct correlations, the outcome indicates tax is largely independent of this relationship.

#### 5.2.5 Financial incentives and Investment feature preferences

Though MIs placed more importance on financial influences, more SIs exclusively chose financially-oriented features to enhance future CE investment vehicles. If MIs typically invest more by virtue of multiple investments, one possible explanation may be that financial factors become increasingly pertinent with total amount invested. This correlation also emerges between amounts invested and financial influences in SIs, lending support to this possibility. If so, this may explain MIs' emphasis on financial influences in addition to non-financial categories, particularly as the ‘preferences’ variable's non-financial measure did not preclude presence of financial features.

### 5.3 Ethical profile

Comparing the four questions taken verbatim from the EMCB scale, mean scores for social and environmental orientations generated by the EMCB's UK sample population (3.4 and 3.19 respectively) [78], are markedly lower than found here (3.87 and 3.77). This indicates this sample is more ethically orientated than average. Further, the new question introduced to reflect the higher environmental propensity anticipated returned the lowest mean. While the scales are not directly comparable, it is notable that the average of all environmental questions was still higher than the UK population's (3.66 compared to 3.22) [78], despite a more demanding question to test environmental orientation.

#### 5.3.1 Environmental influences

Wider environmental influences overwhelmingly appear the principal influence on all investors. Indeed, it was the only influence every investor answered and 97.7% deemed it a positive influence. As such, no significant differences presented. This bias towards environmental orientation is also evidenced in the framework, with the highest-ranking for each group. Overall, NIs held the largest environmental profile. Though the literature proved mixed regarding dominant motivating factors, the findings according to this framework align with Holstenkamp and Kahla [32] and Radtke [31].

Comparing orientation and influences produces an interesting discrepancy between social and environmental categories as social orientation means are higher across all groups. However, this pattern mirrors the UK population sample. Differences between sample means also reveal a comparatively greater environmental orientation overall. Further, standard deviations in every comparable variable indicate lower variation in this sample, implying a more unified response.

#### 5.3.2 Social influences

Community autonomy/independence excepting, the only socially-related differences presented between LIs and NIs concerned more proximal benefits (local economic and social benefits). Notably, however, NIs still rated these as having a slight influence on their investment decision. This is plausibly an indication of higher social orientation - higher than reflected in the value given per local influence, as NIs wish to effect social improvements in unrelated areas. Small-to-medium correlations between EMCB score and local social and economic benefits respectively, partially support this notion, particularly as LIs mirror these with smaller correlations.

This represents a difficulty with translating values for different purposes on the framework, and NIs’ social and COI orientations are likely undervalued as a result. However, simply removing the COP category for NIs was preferred over redistributing then weighting category values, to provide a purer representation of data collected.

#### 5.3.3 Socially responsible investors

The proportion of respondents holding other positive IS appears high at 70%, particularly when contrasted with indications of 20% nationally [44]. This is to be expected, however, as CE constitutes a subset of SR investments. Interestingly, though 74% of MIs held SR IS compared to 64% SIs, no significant difference was detected, and a higher proportion of SIs held these other SR IS prior to CE.

#### 5.3.4 Unproven low carbon technologies

At 45.7%, nearly half expressed a preference for opportunities to invest in unproven, immature RE technologies. As a risky investment, this preference becomes increasingly incompatible with financial motivations. Instead, it likely reflects desire to develop RE technology, indicating a more dominant environmental orientation. Indeed, Bauwens [28] refers to this behaviour as belonging to ‘innovators’ and ‘early adopters’ of institutional innovations, inherent amongst the idealistic and norm-driven. Statistical differences here only presented between SIs and MIs, with MIs more inclined to express this preference. When disaggregated further, statistical differences between SLIs and multi-local investors (MLIs) remain, while NIs are equally divided.

### 5.4 Community profile

Overall, community orientation ranks third on the framework, though this splits to third and fourth when disaggregated into COI and COP respectively. COI factors appeared more prominent for all sets, including LIs.

#### 5.4.1 CEI identification

Unlike SIs and MIs, regional investors differed in extent of identification with their CEIs. Moderate correlations between CEI and COP identification in LIs may indicate that COP factors overlap with and subsequently deepen CEI identification.

#### 5.4.2 Community identification and trust

The literature posited trust and social-norms as core components of social identification, for instance, with COP or CEI [28,60,64,66]. While trust in CEIs to manage financial, social and environmental goals appear equally relevant to each group, there are some underlying differences in the relationships with COP identification. Highly-identifying LIs exhibit greater trust in CEIs to manage ethical and financial goals. However, when LIs are disaggregated into SLIs and MLIs, COP identification in the latter correlates (positively) with the management of ethical goals only, while SIs only do so with financial goals. This implies that with greater COP identification, MLIs were more likely to invest due, in part, to confidence that the CEI will achieve its ethical goals, but this identification did not translate into confidence in investment management. As the opposite is true for SIs, this may reflect the framework results, particularly concerning MIs’ greater ethical orientations.

With regard to trust and identification with respondents’ first CEIs, the more SLIs express identification, the more influenced they were by trust to manage ethical and financial goals. By contrast, NIs and MIs express a similar correlation but for ethical goals only. Again, these are the groups producing greater ethical orientations, which is an observation of note. At the very least, it appears trust may partially account for the relationship between social identification and group characteristics.

### 5.5 Group differences

#### 5.5.1 Regional group differences (financial profile)

When filtered for statistical significance, LIs and NIs differed most in financial orientation. However, the only factors of difference referred to proportions with additional SR IS and, unsurprisingly, the influence of local economic benefits, which are clearly more in LIs’ interests. The former may reflect a higher proportion of investors whose CE investments were prompted when opportunity presented in their locality, rather than an existing predisposition towards SR investments.

#### 5.5.2 Behavioural group differences (financial profile)

SIs and MIs displayed greater variation with differences in comparative risk perception (shares), financial influences and exclusively financially-related preferences. MIs’ propensity to invest despite perceiving CE as riskier than SIs presents an interesting discrepancy. This could reflect a greater ethical orientation as framework outcomes suggest, linking to greater risk tolerance as found in the literature [42,44], but could also be in line with general investment behaviour. Indeed, if considered less risky than conventional alternatives, it follows that MIs may continue to invest in CE.

#### 5.5.3 Other influences

LIs' disposition towards local benefits is unsurprising given their proximal impacts. Interestingly, community autonomy/independence was equally valued by both sub-groups. This may indicate a wider social and environmental interest by NIs, for instance, the political idealism of devolution from powerful actors such as energy companies or government [31,58,68,91]. Further, this was one of several locality-oriented influences uncorrelated to COP identification. Conversely, LI *and* NI CEI identification correlated with the locally-oriented influences unrelated to COP identification. This possibly belies a relationship between wider social influences and CEIs’ ethical COI characteristics.

Locally-oriented influences correlating most with COP identification were community participation and building. Arguably, as the influences requiring the most direct input from members, those who identify most with their COP may be more likely to derive pleasure from such contributions, congruent with hedonic motivations as noted by Dóci and Vasileiadou [25].

#### 5.5.4 Other interested investors known

The interesting observation here is the relative influence of COI. It is likely that local projects attract not only those with COP interests, but with COI interests also. LIs were more likely to know other people interested in participating within their COP, and of those, likely knew twice as many people as NIs did within their ethical COIs. This could be, in part, due to different community characteristics between COP and COI, or that LIs have a larger pool of peers if engaged with both community types (for instance, knowing other COP peers who share similar COIs). Indeed, a small but significant positive correlation between others known outside the COP and ethically-related COI engagement which mirrors a similar correlation for NIs (others known inside COI and ethical COI engagement) lends support to this possibility. Further, a similar correlation between COP and ethical COI engagement could indicate sociability as a positive engagement factor [47]. As posited by Hoffman and High-Pippert [29] and Reinsberger and Posch [58], this link may lie with greater exposure to word-of-mouth and social norm effects. Additionally, the higher proportion of LIs overall (55%) supports Bauwens’ [28] assertion that due to social norm effects, COPs command greater rates of investments.

It is notable that in both communities however, many knew no interested others, which implies ethically-related word-of-mouth and social-norms are less likely to explain first investment motivations for these individuals. However, absence does not necessarily indicate a lack of COI engagement – it is plausible that COI peers were known but not interested in participating. COP has a narrower scope than COI; interests are bounded by location, concentrating opportunities to participate to the CEI in question. By contrast, COI casts a wider net, so peers may be engaged with CE elsewhere, or hold different ethical interests while still facilitating related social norms. Other possibilities could include the effects of alternative non-ethically related COIs, in addition to other factors yet to be identified.

### 5.6 Theoretical discussion on multiple investor dynamics

Lindenberg and Steg [71] asserted that exposure to environmental social norms promotes normative goal frames through the erosion of gain and hedonic goal frames effects and that when examined in the sustainability context, normative goal frames suggest acting in a pro-environmental way, while gain and hedonic goal frames frequently lead to not acting in an eco-friendly manner. Hence, the Normative Goal Frames such as actions driven by environmental and ethical objectives primarily motivates the first investments of investors of multiple community energy investments and not Gain Goal Frame (such as financial return) and a Hedonic Goal Frame (such as seeking positive feeling).

Contrasting MIs’ influence ratings from their first and subsequent investments present a dramatic change, which appears to support this theory (Fig. 6). The only unaltered influence was wider environmental, which is likely due to being maximally rated for the first investment; certainly, its influence did not diminish. Of the remainder, all produced a statistically significant increased effect. Further, the proportion of all investors holding other SR savings or investments prior to their first CE investment (41%) subsequently grew to 70%.

Social norm theory offers a compelling explanation for these distinctive findings. Referring to Bamberg, Rees and Seebauer [26] and the internalisation of CEI group norms over time, it is plausible that respondents became increasingly aware of the beneficial impacts resulting from their investments, bolstering efficacy-beliefs and in turn, extending socially responsible behaviours. Additionally, MIs indicated that experience fostered greater trust, which elicited a ‘moderate influence’ on subsequent investment decisions. Moreover, these effects are not limited to the environmental category, but also community, social and financial effects. The personal financial influence was not assessed beyond first investment, though could possibly explain the higher rating afforded by MIs, as awareness of the financial benefits may also have increased over time.

Interestingly, COP-related factors increased most and significantly. Though for NIs, unless all subsequent investments were in local projects, some of these may technically be COI-related, or instance, ‘COP figureheads’ local to the CEI but not the investor. The medians were unanimous across all multi-investors except for community participation, which received a higher value from NIs (though no significant difference presented). This unexpected development regarding COP requires clarification from participants regarding their interpretations of COP influences. The next greatest increase presented in social, followed by COI. The least affected was financial orientation, which also accounted for differences in conventional-only investors.

The main differences lie between LIs and NIs. NIs are more likely to be SR investors, which amounts to a greater proportion demonstrating SR investment behaviours prior to CE. The role of trust is significant for NIs regarding ethical goals, as opposed to financial goals for LIs, and they participate despite weaker prompting from COI sources. These behaviours are likely the result of those who seek out opportunities to generate social good in addition to (or perhaps regardless of) financial returns. Indeed, NI is the only group which displays a statistically significant negative correlation between environmental orientation and financial influences, (which disaggregation subsequently attributes to MNIs). By contrast, COP factors predominantly (though not exclusively) prompt LIs to participate, with the added attraction of financial and ethical benefits.

This is not to undermine ethical orientations of LIs, however. Indeed, these also recorded above-average ethical orientations and some will likely have participated through ethical orientation regardless of, or in addition to regional-affect. Differences in investment behaviour may follow the recruiting community. For instance, NIs are recruited through COI and predominantly adopt repeat invest behaviours. With regards to LIs, COI and COP appear to take the role of complementary support mechanisms to CEIs, with COP an effective recruiting mechanism and COI (via CEI), an effective route towards repeat investments and heightened awareness of environmental and social issues. This appears true for the 50% of LIs who became MIs, and so likely become non-local to subsequent projects.

## 6 Conclusions

This study sought to identify the factors underpinning Community Energy (CE) investor characteristics and motivations within the UK context, to inform suitable investment models in absence of state-supported financial mechanisms. The literature illustrated a heterogeneity of CE models, producing an array of incentives which, in turn, attract a broad spectrum of investor types. The study adds to this existing knowledge by capturing differences among investors with differing regional affect and investment behaviours. The study also addresses the demographic dynamics of UK CE investors.

Overall, the findings indicate that though differences are noted, this sample is not excessively heterogeneous. This is particularly so regarding the demographic profile, with little variation between groupings. The profile is striking with high proportions of males, and older, wealthier individuals with typically high-level education and career experience. Instead, differences are largely exposed through socio-psychological profiling against a conceptual framework. Results indicate that investors are predominantly ethically-oriented, particularly toward environmental concerns. The implications of this is that, whiles the motivation for CEIs from individual investors may be ethically driven, CEIs generates co-benefits, which are tangible and delivers impactful changes to the economy in a forward-thinking manner compared to traditional investments. This is because, a significant portion of investment raised for new projects are usually spent locally; thus, boosting local economies. These socio-economic co-benefits are derived as CEIs holds the promise of an economy and society based on co-operation rather than competition, within the boundaries of environmental sustainability.

Community and social factors also appear to play significant roles in investor participation while financial orientation is least dominant. In combination, this gives the impression of elasticity regarding financial incentives; somewhat riskier or less lucrative returns would unlikely present a death knell to the CE sector. However, financial factors are still relevant and such changes should disincentivise more financially-oriented investors. In some ways, the findings are no surprise: those recruited solely through COP factors are less inclined to make repeat investments, though they express interest in additional CE projects eliciting regional affect. Conversely, COI-sourced respondents are more likely to make repeated investments and do so regardless of locality. These orientations combine to produce greater proportions of non-locals amongst repeat investors and locals amongst one-time investors.

Arguably, these numbers could be explained by the geographic nature of COP and its constraints on creating additional investment opportunities for single local investors. However, financial incentives and potentially COI appear to play additional roles in determining repeat behaviours amongst local respondents. Findings indicate SIs may have responded more to the loss of now-defunct state-supported incentives, potentially explaining their disinclination to invest again. By contrast, LIs who transitioned from single into MIs indicate their loyalty to CE may primarily be related to ROI without emphasis on FiT. As SLIs also present a similar relationship with ROI, we can assume that ROI alone is insufficient to compel further investment. For MNIs, any relationship between valued financial incentives and loyalty-related effects of their performance was undetectable. As MIs and NIs typically present as most ethically-oriented, it is plausible these investors are more motivated by non-financial factors.

Overall, these findings suggest MNIs, the largest grouping at 31.9%, are least affected by cuts and capping of FiT or reductions in ROI, though not necessarily at all costs. MLIs are attracted via COP but incentivised beyond it, which may be partially reflected in financial orientation, but COI likely plays a role in reinforcing wider ethical orientations for many. This was particularly evidenced in adjustments to influence-ratings from initial to ongoing investments. Indeed, all investors identify as more ethically-oriented than the general UK population, though non-locals present as most ethically and COI-oriented. SLIs typically identify more with their COP and CEI than MLIs and NIs respectively. Whether ROI must be more lucrative or secure, whether alternative incentives such as those relating to COP, COI or ethical factors should be buttressed to inspire repeat investment behaviours, or what combination of these is optimal requires more research. This is true for both SNIs and SLIs, though COI and COP-related factors are defensibly worthwhile focal areas to begin.

Improved understanding of investor characteristics, orientations and the socio-psychological mechanisms, which facilitate and perpetuate participation could help CEIs target investors more effectively. For instance, by leveraging the complementary forces of COI and COP to target, engage and service investors accordingly. Of local investors, likely predominantly recruited through COP, half go on to make repeat investments. Though financial incentives likely play a role in repeat investments, CEI-induced COI effects appear to play a substantial role in effecting this behaviour. The findings support the notions of heightened trust, efficacy beliefs, and exposure to CEI group norms as underlying mechanisms. As such, COI may not just offer a source of recruitment but for some investors but may also be a transformation mechanism.

Further research is recommended into the relationship between region-specific factors and CE investor characteristics is an area ripe for further research. Through the lens of local and non-local investors, this study addressed region within the context of CEI proximity, affect and identification effects of community. However, more granular research covering socio-political, socio-economic and institutional logic would help to ascertain the impact of more circumstantial influences to complement this investor-centric approach. Additionally, this study generated an unexpected finding in comparative risk perception between CE and conventional investments which could be explored further. Indeed, whiles single investors (SIs) and multi investors (MIs) displayed greater variation between them with differences in comparative risk perception, financial influences and exclusively financially-related preferences, the behaviour contradicts that of traditional investors with respect to risk perception. This contradiction stems from the fact that traditional finance theory posits that sustainable investments, such as in CEI, would only be considered if they are equally attractive as other investments in terms of risk and returns. MIs’ propensity to invest for instance despite perceiving CE as riskier goes against the traditional finance theory. Whiles the paper speculates that this could reflect a greater ethical orientation as the results of the framework outcomes suggest, the paper suggests that for further research, this contradiction should be further examined.

Community energy initiatives (CEIs) are built on principles aligned to the general energy policy framework of the Energy Trilemma, which seeks to define the need to find balance between three distinct areas of the energy sector; namely, energy security, energy equity, and sustainability and its impact on everyday lives. Specifically, it is noted that the general benefits of CEIs are wide, ranging from increased energy security through lower energy costs and greater price certainty to an accelerated access to renewable energy through citizen-driven innovation and a much broader participation in the energy system. The attain and sustain these benefits, CEIs are dependent on important levers, central among these being policy levers needed to overcome inherent barriers in the sector. Globally, Feed-In Tariff has been an important and very effective policy instrument in supporting renewable energy market growth including for CEIs. This is mainly due to its non-discriminatory design and administration and its use an economic incentive to grow the renewable energy industry. In the UK, changes introduced back in 2016 to effectively cap the level of deployment for each eligible technology under the Feed-in Tariffs, in an attempt reduce annual expenditure, is seen as weakening policy support for the sector. This is because, the introduction of the caps has greatly hindered the installation of new renewable energy systems under the Feed-in Tariffs.

## Ethical consideration

We confirm that any aspect of the work involving human patients was conducted with ethical approval granted by the Kent Business School, University of Kent Research Ethics Committee. In addition, informed consent was obtained from all respondents prior to their participation in completing the questionnaires.

## CRediT authorship contribution statement

**Helen Mullen:** Writing – original draft, Resources, Methodology, Investigation, Formal analysis, Data curation, Conceptualization. **Charles Turkson:** Writing – original draft, Supervision, Methodology, Formal analysis, Data curation, Conceptualization. **Adolf Acquaye:** Writing – original draft, Writing – review & editing, Supervision, Resources, Methodology, Formal analysis, Conceptualization.

## Declaration of competing interest

The authors declare that they have no known competing financial interests or personal relationships that could have appeared to influence the work reported in this paper.

## Figures and Tables

**Table 2:** Survey Design- CE Investment Section

CE INVESTMENT SECTION			
Categories	Variable	Levels	Data Collection
CEI Investment Details	CEI name(s)		Select and/or free text
	Shares/bonds	Shares, bonds, both	Select one
	Proximity/community attachment	Local, Non-Local	Select one
	Investment amount	For each CE Investment declared	Banded amounts up to investment maximum (£100,000)
Community Factors (based on local/non-local identification)	CEI Investment- source of awareness	COP, COI and Other options provided	Select one & ‘Other' text
	Interested others known	Inside and outside COP; Inside and outside COI	Free text
	CEI role	General member or board member	Select one
Community, social and environmental influences	Investment influences	Recommendations from COP peers	5- point Likert scale (opt out)
		Recommendations from COP figureheads	
		Recommendations from COI peers	
		Community building	
		Community participation	
		Community autonomy/independence	
		Local social benefits	
		Local economic benefits	
		Local environmental benefits	
		Wider environmental benefits	
		Greater trust in CEI to manage investment	
		Greater trust in CEI to manage ethical goals	

**Table 3:** Survey Design- Financial Section

FINANCIAL SECTION			
Categories	Variable	Levels	Data Collection
Financial Attitude	Financial influences	Return on Investment	5- point Likert scale (opt out)
		Tax relief	
		Feed-in-Tariff	
		Portfolio Diversification	
	Comparative risk perception (conventional vs. SR vs. CE)	Shares, Bonds	Likert Rating Scale 1-6
Investment behaviors	Types of savings/investments held	Conventional and SR	Select one
	Amount of savings/investments held		Banded amounts: £0-£250,000+
	When began SR investing	SR Investors only; Before or after first CE investment	Select one

**Table 4:** Survey Design- Demographic Section

DEMOGRAPHIC SECTION			
Categories	Variable	Levels	Data Collection
Demographic Profile	First-half Postcode		Text
	Birth year		
	Gender		Select one (opt out)
	Employment Status		Select one
	Occupational Status		
	Annual Net-income		
	Qualification levels		
	Home ownership		
	Newspapers Read		

**Table 5:** Survey Design- Ethical Section

ETHICAL SECTION			
Categories	Variable	Levels	Data Collection
Loyalty to CE investments	Level of discouragement in future CE investments if existing CEI underperformed on certain goals	Financial goals	7-point Likert scale
		Social goals (CEI social fund or indirect benefits)	
		Environmental goals	
Social orientation	Pro-social consumer behaviours	Boycotting socially irresponsible firms	5-point Likert scale
		Boycotting firms due to labour exploitation	
		Pay more for SR products	
Environmental orientation	Pro-environmental consumer behaviours	Choose least environmentally damaging products	
		Switched products for environmental reasons	
		Boycott environmentally damaging products	
		Boycott harmful household products	
		Purchase and dispose of products according to recycling credentials	
		Engage in less mainstream environmental behaviours	
Ethical COI engagement	Level of engagement with COI peers	Social	
		Environmental	
Community identification	Extent of identification with COP or CEI	COP (local only); CEI (all)	5- point Likert scale
Future investments	Preferences requested	Range of options, categorised as follows:	Select multiple
		Financial (investment-term options)	
		CEI-type preferences (unproven RE tech, new or existing projects)	
		Environmental (CEI with unproven technology)	
	Intention to make more CE investments		5-point Likert scale
	CE investment amount intended within next year		Banded amounts up to invest. Max. (£100,000)
	Ethical returns required from future CE and SR investments (broken down by local and non-local CEI options)	Wider and/or local: Social returns, Environmental returns	Select

## Footnotes

1 Where stakeholders possess stronger decision-making powers compared to centralised provision.

2 Degrowth is defined as improving human and ecological wellbeing by equitably reducing production and consumption, both locally and globally.

3 regulatory and organisational.

4 social-norms and routine practices.
