# Supplementary material for: An examination of motivation factors driving investor behaviours towards socially responsible community energy initiatives
Source: Heliyon. 2024 Mar 6;10(5):e27490. doi: 10.1016/j.heliyon.2024.e27490 (PMC10943446; doi:10.1016/j.heliyon.2024.e27490)
Supplement: Multimedia component 1 [file mmc1.docx]

**“An Examination of** **Motivation Factors driving Investor Behaviours towards Socially Responsible Community Energy Initiatives.**

**SUPPLEMENTARY INFORMATION**

**Supplementary Table 1: Survey Design- CE Investment Section**

| ***CE INVESTMENT SECTION*** | | | |
| --- | --- | --- | --- |
| ***Categories*** | ***Variable*** | ***Levels*** | ***Data Collection*** |
| CEI Investment Details | CEI name(s) |  | Select and/or free text |
|  | Shares/bonds | Shares, bonds, both | Select one |
|  | Proximity/community attachment | Local, Non-Local | Select one |
|  | Investment amount | For each CE Investment declared | Banded amounts up to investment maximum (£100,000) |
| Community Factors (based on local/non-local identification) | CEI Investment- source of awareness | COP, COI and Other options provided | Select one & 'Other' text |
|  | Interested others known | Inside and outside COP; Inside and outside COI | Free text |
|  | CEI role | General member or board member | Select one |
| Community, social and environmental influences | Investment influences | Recommendations from COP peers | 5- point Likert scale (opt out) |
|  |  | Recommendations from COP figureheads |  |
|  |  | Recommendations from COI peers |  |
|  |  | Community building |  |
|  |  | Community participation |  |
|  |  | Community autonomy/independence |  |
|  |  | Local social benefits |  |
|  |  | Local economic benefits |  |
|  |  | Local environmental benefits |  |
|  |  | Wider environmental benefits |  |
|  |  | Greater trust in CEI to manage investment |  |
|  |  | Greater trust in CEI to manage ethical goals |  |
| **Supplementary Table 2: Survey Design- Financial Section**   \| ***FINANCIAL SECTION*** \| \| \| \| \| --- \| --- \| --- \| --- \| \| ***Categories*** \| ***Variable*** \| ***Levels*** \| ***Data Collection*** \| \| Financial Attitude \| Financial influences \| Return on Investment \| 5- point Likert scale (opt out) \| \|  \| Tax relief \| \|  \| Feed-in-Tariff \| \|  \| Portfolio Diversification \| \|  \| Comparative risk perception (conventional vs. SR vs. CE) \| Shares, Bonds \| Likert Rating Scale 1-6 \| \| Investment behaviors \| Types of savings/investments held \| Conventional and SR \| Select one \| \| Amount of savings/investments held \| Banded amounts: £0-£250,000+ \| \| When began SR investing \| SR Investors only; Before or after first CE investment \| Select one \| | | | |

**Supplementary Table 3: Survey Design- Demographic Section**

| ***DEMOGRAPHIC SECTION*** | | | |
| --- | --- | --- | --- |
| ***Categories*** | ***Variable*** | ***Levels*** | ***Data Collection*** |
| Demographic Profile | First-half Postcode |  | Text |
|  | Birth year |  |  |
|  | Gender |  | Select one (opt out) |
|  | Employment Status |  | Select one |
|  | Occupational Status |  |  |
|  | Annual Net-income |  |  |
|  | Qualification levels |  |  |
|  | Home ownership |  |  |
|  | Newspapers Read |  |  |

**Supplementary Table 4: Survey Design- Ethical Section**

| ***ETHICAL SECTION*** | | | |
| --- | --- | --- | --- |
| ***Categories*** | ***Variable*** | ***Levels*** | ***Data Collection*** |
| Loyalty to CE investments | Level of discouragement in future CE investments if existing CEI underperformed on certain goals | Financial goals | 7-point Likert scale |
|  |  | Social goals (CEI social fund or indirect benefits) |  |
|  |  | Environmental goals |  |
| Social orientation | Pro-social consumer behaviours | Boycotting socially irresponsible firms | 5-point Likert scale |
|  |  | Boycotting firms due to labour exploitation |  |
|  |  | Pay more for SR products |  |
| Environmental orientation | Pro-environmental consumer behaviours | Choose least environmentally damaging products |  |
|  |  | Switched products for environmental reasons |  |
|  |  | Boycott environmentally damaging products |  |
|  |  | Boycott harmful household products |  |
|  |  | Purchase and dispose of products according to recycling credentials |  |
|  |  | Engage in less mainstream environmental behaviours |  |
| Ethical COI engagement | Level of engagement with COI peers | Social |  |
|  |  | Environmental |  |
| Community identification | Extent of identification with COP or CEI | COP (local only); CEI (all) | 5- point Likert scale |
| Future investments | Preferences requested | Range of options, categorised as follows: | Select multiple |
|  |  | Financial (investment-term options) |  |
|  |  | CEI-type preferences (unproven RE tech, new or existing projects) |  |
|  |  | Environmental (CEI with unproven technology) |  |
|  | Intention to make more CE investments |  | 5-point Likert scale |
|  | CE investment amount intended within next year |  | Banded amounts up to invest. Max. (£100,000) |
|  | Ethical returns required from future CE and SR investments (broken down by local and non-local CEI options) | Wider and/or local: Social returns, Environmental returns | Select |
